# Enhancement of dendritic persistent Na^+^ currents by mGluR5 leads to an advancement of spike timing with an increase in temporal precision

**DOI:** 10.1186/s13041-018-0410-7

**Published:** 2018-11-09

**Authors:** Weonjin Yu, Jong-Woo Sohn, Jaehan Kwon, Suk-Ho Lee, Sooyun Kim, Won-Kyung Ho

**Affiliations:** 1 Department of Physiology, Seoul National University College of Medicine, 103 Daehak-ro, Jongno-gu, Seoul, 03080 Republic of Korea; 2 Neuroscience Research Institute, Seoul National University College of Medicine, Seoul, 110-799 Republic of Korea; 3 Department of Biological Sciences, Korea Advanced Institute of Science and Technology, Daejeon, 305-701 Republic of Korea

**Keywords:** mGluR5-dependent plasticity, Persistent sodium current, CA1 pyramidal neurons, Intrinsic excitability

## Abstract

Timing and temporal precision of action potential generation are thought to be important for encoding of information in the brain. The ability of single neurons to transform their input into output action potential is primarily determined by intrinsic excitability. Particularly, plastic changes in intrinsic excitability represent the cellular substrate for spatial memory formation in CA1 pyramidal neurons (CA1-PNs). Here, we report that synaptically activated mGluR5-signaling can modulate the intrinsic excitability of CA1-PNs. Specifically, high-frequency stimulation at CA3-CA1 synapses increased firing rate and advanced spike onset with an improvement of temporal precision. These changes are mediated by mGluR5 activation that induces cADPR/RyR-dependent Ca^2+^ release in the dendrites of CA1-PNs, which in turn causes an increase in persistent Na^+^ currents (I_Na,P_) in the dendrites. When group I mGluRs in CA1-PNs are globally activated pharmacologically, afterdepolarization (ADP) generation as well as increased firing rate are observed. These effects are abolished by inhibiting mGluR5/cADPR/RyR-dependent Ca^2+^ release. However, the increase in firing rate, but not the generation of ADP is affected by inhibiting I_Na,P_. The differences between local and global activation of mGluR5-signaling in CA1-PNs indicates that mGluR5-dependent modulation of intrinsic excitability is highly compartmentalized and a variety of ion channels are recruited upon their differential subcellular localizations. As mGluR5 activation is induced by physiologically plausible brief high-frequency stimulation at CA3-CA1 synapses, our results suggest that mGluR5-induced enhancement of dendritic I_Na,P_ in CA1-PNs may provide important implications for our understanding about place field formation in the hippocampus.

## Introduction

The hippocampus is a major part of the brain responsible for spatial learning and memory. When animals navigate large environments, a subset of hippocampal pyramidal neurons termed ‘place cells’ encode the spatial information. These cells exhibit location-specific increases in firing frequency when the animal traverses a specific location known as a place field [18]. Understanding of the underlying mechanisms that contribute to the activity-induced modulation of firing frequency is therefore critical for deciphering the processes of hippocampal memory formation.

Plasticity in the hippocampus has been strongly implicated in spatial memory formation [22, 27]. Much research has been devoted to synaptically induced plasticity, which involves fast ionotropic glutamate receptors, such as *N*-methyl-*D*-aspartate (NMDA) and α-amino-3-hydroxi-5-methyl-ioxyzole-4-propionic acid (AMPA; [12]). However, recent attention has focused on the involvement of modulation of intrinsic excitability in the formation of place field in CA1 pyramidal neurons (CA1-PNs; [2, 14, 22, 23]). During spatial navigation, CA1-PNs that later become place cells showed a lower AP threshold and an increased likelihood of firing [2]. These results imply the importance of the modulation of the components that influence the firing ability of CA1-PNs.

Metabotropic glutamate receptors (mGluRs) are widely expressed in the brain and play an important role in the regulation of membrane excitability, synaptic transmission and network activity [1, 3, 17]. Among many groups of mGluRs (Groups I, II, and III mGluRs), the group I mGluRs, which comprise mGluR1 and mGluR5, are especially implicated in the control of intrinsic excitability in hippocampal neurons. The activation of the group I mGluRs are coupled to G_q_ protein-mediated Ca^2+^ signaling pathways, which ultimately modulate various ion channels that shape the input-output relationship of neuronal network. We have recently demonstrate that high-frequency stimulation (HFS) of the Schaffer collateral pathway leads to the reliable activation of postsynaptic mGluR5 in CA1-PNs, producing the short-term potentiation of EPSP-to-spike (E-S) coupling [32]. We showed the involvement of persistent Na^+^ currents (I_Na,P_) in proximal apical dendrites in mGluR5-dependent E-S potentiation, but the role of dendritic I_Na,P_ enhancement in intrinsic excitability and firing properties in CA1-PNs remains to be characterized.

In this study, we provide the novel mGluR-mediated mechanisms of modulating firing property of CA1-PNs. Our results revealed that the effects of mGluR5 activation in response to HFS to SC pathway are different from those of global mGluR5 activation. Selective modulation of I_Na,P_ in the proximal apical dendrites of CA1-PNs leads to increased firing frequency, advancement of spike timing, and improvement of temporal precision during behaviorally relevant oscillation. Given that mGluR5 can be activated by physiologically relevant network condition [5], we propose that the activation of mGluRs may represent a key cellular mechanism for determining place cell firing.

## Results

### HFS-induced mGluR activation causes increases in AP firing and its temporal precision

**Fig. 1:**
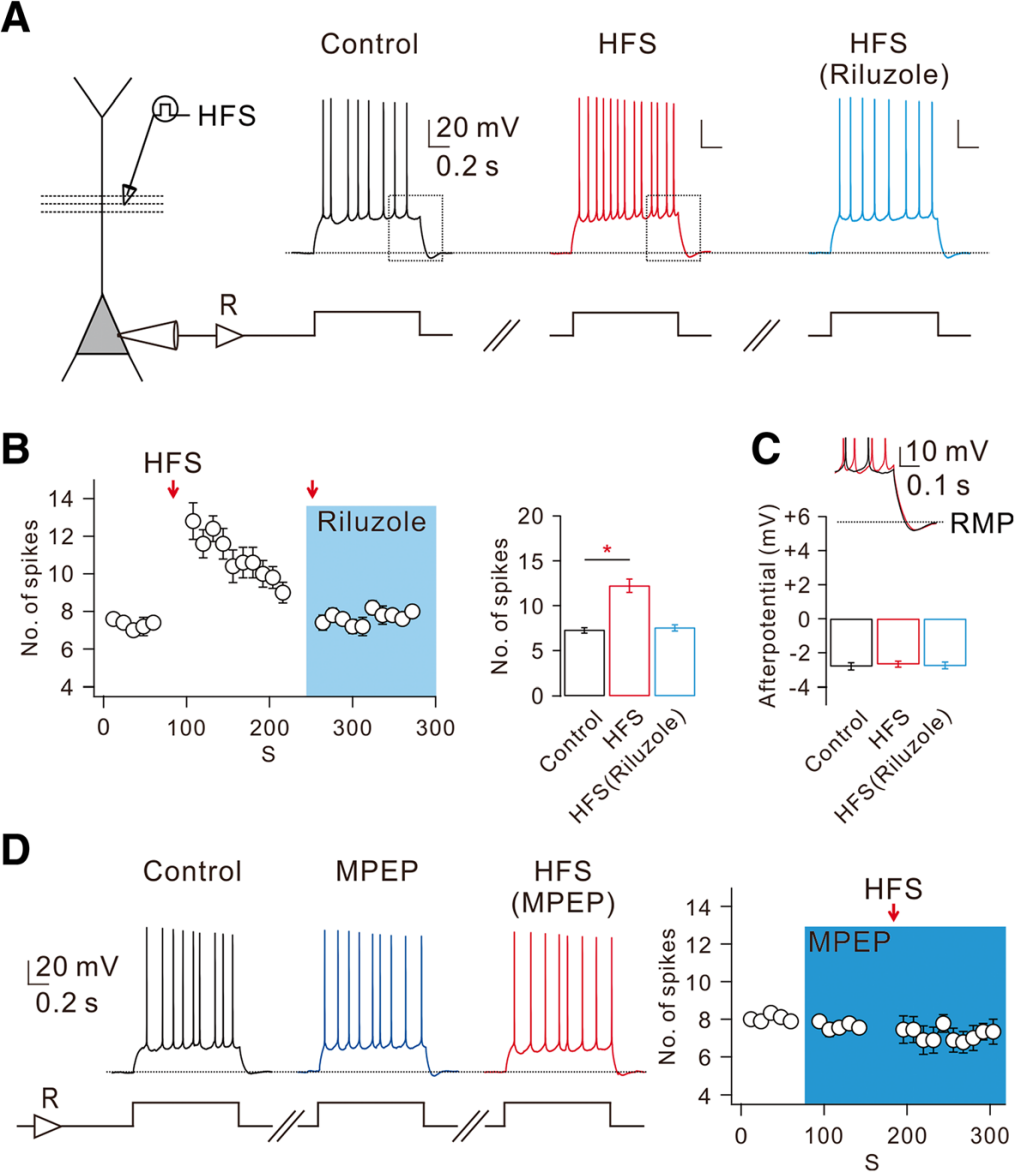
mGluR5-dependent increased CA1 neuronal excitability is mediated by enhancement of I_Na,P_. **a** Left, schematic diagram illustrating the recording configuration showing somatic current-clamp recording (CC) with extracellular electrodes positioned to stimulate SC inputs. Right, representative traces demonstrated HFS-increased number of spikes in response to square current injections before HFS (black), after HFS (red), and after HFS in riluzole (blue, 10 μM). **b** Left, number of spikes evoked by somatic current injections was counted and plotted against time (*n* = 5). Right, pooled data of the mean number of spikes during somatic current injection obtained for first 1 min in each condition (*n* = 5, control vs HFS, *P < 0.05). **c** Top, inset shows superimposed view corresponding to the box shown in (**a**). Bottom, pooled data of the afterpotential following square currents injections in different conditions (*n* = 5). **d** Left, representative traces demonstrate the effects of MPEP (10 μM) on HFS-induced changes of number of spikes. Right, summary plots of the mean number of spikes evoked by somatic current injections as a function of time (*n* = 9)

We recently reported that high-frequency stimulations (HFS; 100 Hz for 0.5 s) to Schaffer Collateral (SC) synapses trigger mGluR-dependent Ca^2+^ release in apical dendrites of CA1-PN, which in turn induce calmodulin-dependent increase in persistent Na current (I_Na,P_) that leads to E-S potentiation [32]. To investigate the effects of dendritic I_Na,P_ increases on intrinsic excitability of CA1-PNs, we tested whether action potential (AP) firing in CA1-PNs evoked by depolarizing current injection is affected by HFS. To rule out any NMDA receptor-dependent changes or the recruitment of GABAergic inhibition, we performed this experiment in the presence of APV (50 μM) and PTX (100 μM) in the bath. We adjusted the current amplitude so that the AP number during 1-s depolarization was between 6 and 8 under control conditions and repeated this stimulation at 10 s interval (Fig. 1a and b). We then applied HFS through a stimulation electrode which was positioned at 80~ 100 μm away from the soma. After HFS to the SC pathway, there was no change in resting membrane potential (RMP; control: − 64.31 ± 0.55 mV, after HFS: − 64.25 ± 0.61 mV, *n* = 8), but AP number increased significantly (Fig. 1a and b). To examine the time course of the HFS effect on intrinsic excitability, we plotted the number of APs as a function of time (Fig. 1b). The increase in AP numbers following HFS did not last long and the increased AP numbers decreased gradually to the basal level within 2 min (Fig. 1b), which is consistent with the effect of HFS on E-S potentiation reported in our previous paper [32].

As a mechanism for HFS-induced increases in intrinsic excitability, we examined the involvement of I_Na,P_ using I_Na,P_ blocker riluzole [30]. In the presence of riluzole (10 μM), HFS-induced increases in AP number were abolished (Fig. 1a and b). These results support the idea that HFS-induced enhancement of dendritic I_Na,P_ facilitates AP firing. We previously showed that HFS-induced enhancement of dendritic I_Na,P_ is mediated by mGluR5 activation [32], and confirmed here that HFS-induced increases in AP number were indeed abolished in the presence of mGluR5 blocker MPEP (Fig. 1d). However, afterhyperpolarization (AHP) following repetitive APs was affected neither by HFS nor by riluzole (Fig. 1a and c).

**Fig. 2:**
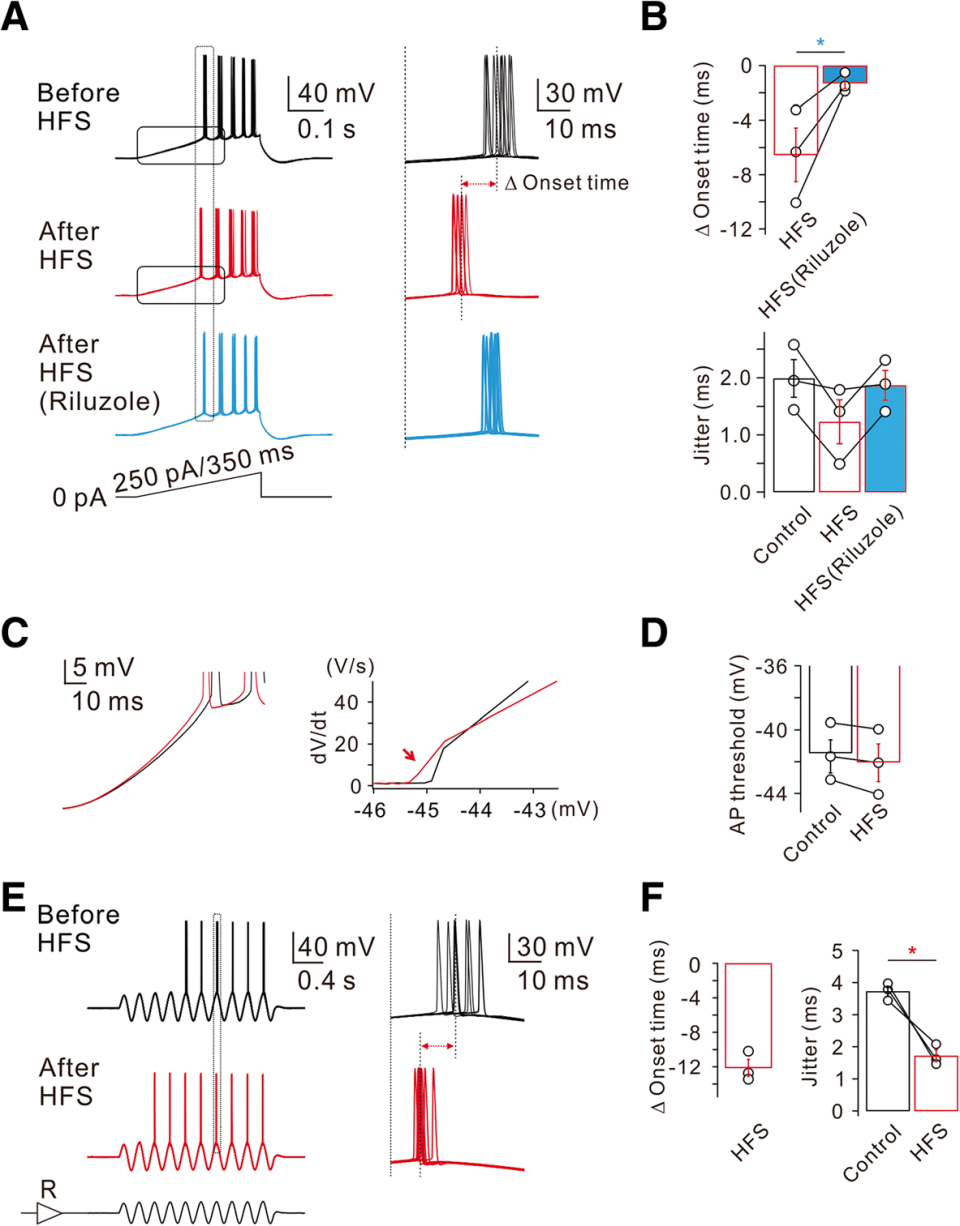
HFS-induced decrease of spike onset timing and jitter. **a** Left, representative traces in response to slow current ramp (250 pA/350 ms) injections before (black), after (red), and after in riluzole (bottom, blue). Right, representative traces demonstrate HFS-decreased spike timing onset and jitter are reversed by riluzole. **b** Pooled data summarizes the effects of HFS and subsequent addition of riluzole on spike onset timing and jitter (Δ onset time, *n* = 3, HFS vs HFS (Riluzole), *P < 0.05). **c** Left, averaged voltage traces from the traces shown in (**a**) before (black) and after (red) HFS are superimposed. Right, phase-plane plots of d*V*/d*t* vs corresponding voltage from the traces shown in (left, **c**) before (black) and after (red) HFS are shown superimposed. Note that the threshold for AP initiation is shifted after HFS in control (red arrow in **c**, *n* = 3; **d**). **e** Left, representative traces in response to sine wave current with theta frequency (5 Hz) via somatic pipette before (black) and after HFS (red). **f** Pooled data summarizes the effects of HFS in response to sine wave currents injection on spike onset timing and jitter (jitter, *n* = 3, control vs HFS, *P < 0.05)

To further investigate the physiological relevance of I_Na,P_ enhancement on neuronal excitability, we examined the effect of HFS on firing activity of APs evoked by a slow current ramp (250 pA/350 ms; Fig. 2). Voltage responses obtained by applying the same ramp for 10 times were superimposed (Fig. 2a), and the effects of HFS on the spike onset time and jitters were analyzed (Fig. 2b). The spike onset time was measured by the duration from the initiation point of the ramp to the first AP. After HFS, spike onset time was advanced by 6.5 ± 2.0 ms (*n* = 3), but this advancement did not occur in the presence of riluzole (Fig. 2a and b). Spike timing jitter, which represents spike timing precision, was quantified as the standard deviation of the spike onset latencies which were measured while the same ramp was applied for 10 times. The spike timing jitter in control was 2.0 ± 0.3 ms (*n* = 3), while it decreased to 1.2 ± 0.4 ms after HFS, and this decrease was also reversed by riluzole (Fig. 2a and b). These results suggest that HFS-induced enhancement of I_Na,P_ not only advances the spike onset but also increases the temporal precision. To investigate how increased I_Na,P_ affect both spike onset and temporal precisions, we further analyzed the depolarizing phase during a slow current ramp, and found that the rate of membrane depolarization exhibited prominent changes on the later phase of slow depolarization (Fig. 2c), which might account for the facilitation of AP generation and a decrease in spike jitter. Somatic spike threshold was slightly hyperpolarized (control: − 41.5 ± 1.0 mV; after HFS: − 42.0 ± 1.2 mV, *n* = 3, Fig. 2d), but there was no statistical significance.

To mimic more physiological voltage responses occurring in vivo [10, 11], we injected an oscillating sine wave current with theta frequency (5 Hz) via somatic pipette, while current amplitudes were adjusted to evoke APs at 5th or 6th sine wave. Representative voltage traces obtained in responses to the injection of 8 sine waves were superimposed (Fig. 2e). After HFS, AP firing was advanced so that 1st AP was evoked at 3rd or 4th sine wave. To examine the effect of HFS on AP onset time, we measured AP onset at 7th sine wave (indicated by a grey box, Fig. 2e). The onset time was reduced by 12.1 ± 1.0 ms and spike timing jitter was also significantly reduced (*n* = 3, Fig. 2f). These results suggest that activity-dependent advancement of AP firing and increased temporal precision may play a role during the realistic network oscillation.

### Group I mGluR-dependent changes in the intrinsic excitability of CA1-PNs

**Fig. 3:**
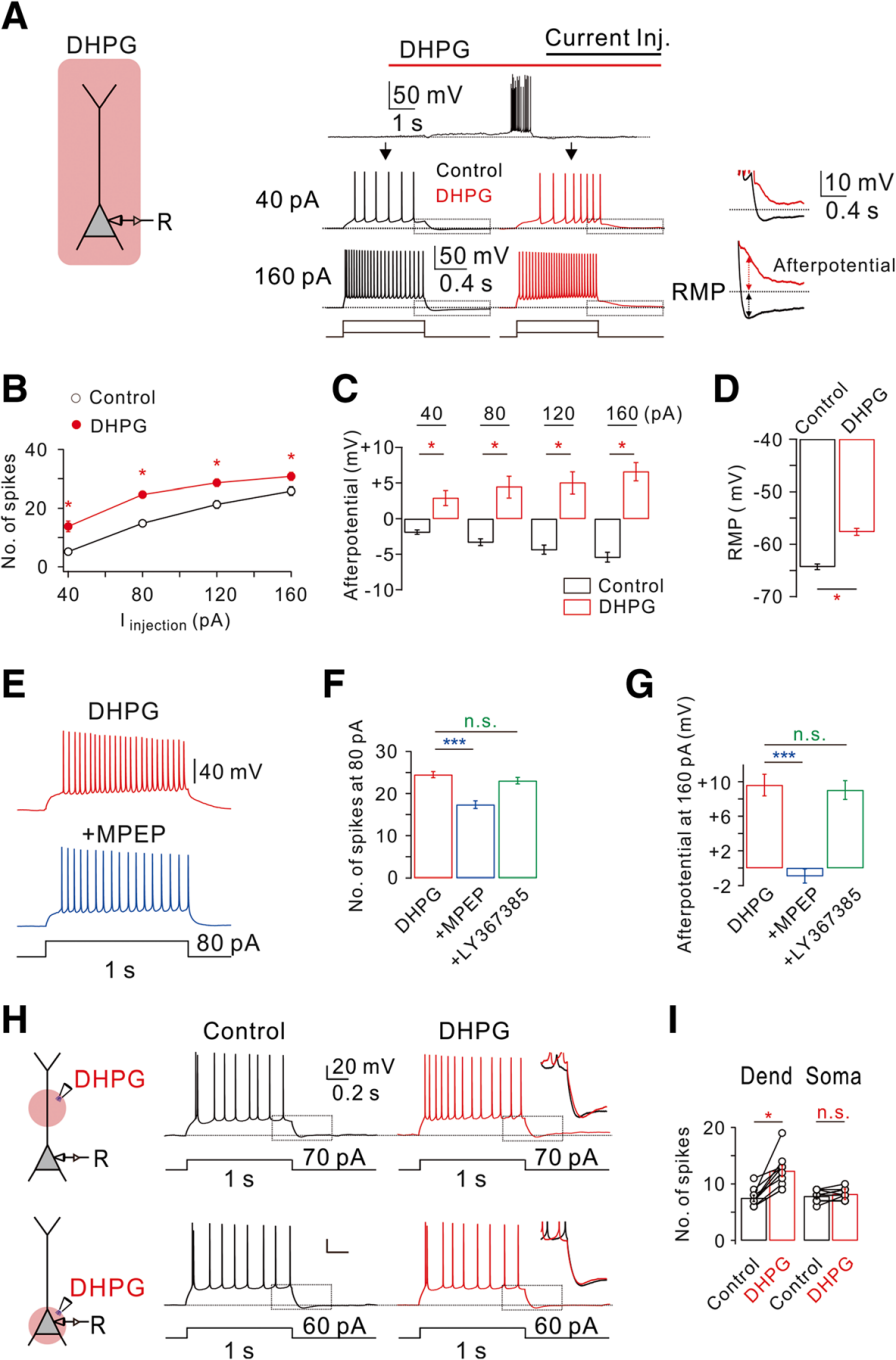
Bath applications of DHPG stimulates mGluR5 to increase hippocampal CA1 neuron excitability. **a** Left, a schematic drawing of experimental condition demonstrates a neuron being recorded with bath applications of DHPG. Right, representative traces demonstrate DHPG-induced depolarization of membrane potential (upper trace) and changes in AP firing frequency and afterpotential in response to square pulse injections (40 pA, 160 pA, lower traces). Note that afterpotentials following 2 different magnitudes of current injections. **b** FI curve summarizes the effects of DHPG on AP firing frequency in response to square current injections (*n* = 16, control vs DHPG, *P < 0.05). **c** Bar graphs summarize the effects of DHPG on afterpotentials following 4 different square current injections (*n* = 5, control vs DHPG, *P < 0.05). **d** Bar graphs summarize the effects of DHPG on resting membrane potentials (*n* = 5, control vs DHPG, *P < 0.05). **e** Representative traces demonstrate the effects of MPEP on DHPG-induced changes of AP firing frequency and afterpotentials. **f** Pooled data of the mean number of spikes following 80 pA square pulse injections in each condition (DHPG, *n* = 14; MPEP, *n* = 14; LY367385, *n* = 16, DHPG vs MPEP, ***P < 0.001; DHPG vs LY367385, n.s., *P* > 0.05). **g** Bar graphs summarize the effects of DHPG and subsequent addition of MPEP or LY367385 on afterpotentials following 160 pA square pulse injections (DHPG, *n* = 7; MPEP, *n* = 10; LY367385, *n* = 7; DHPG vs MPEP, ***P < 0.001; DHPG vs LY367385, n.s., *P* > 0.05). **h** Left, a schematic drawing of experimental condition demonstrates a neuron being recorded with dendritic or somatic local applications of DHPG (250 μM). Right, representative traces demonstrate the effects of local application of DHPG on AP firing frequency and afterpotentials. Inset shows an expanded view corresponding to the box shown in the voltage traces. **i** Pooled data of the mean number of spikes during somatic current injection in each condition (Dend, *n* = 10; Soma, *n* = 11; Dend, Control vs DHPG, *P < 0.05; Soma, Control vs DHPG, n.s., *P* > 0.05)

It is well known that group I mGluR activation affects intrinsic excitability in a variety of neuronal types in the brain [6, 24], including hippocampus [26]. To compare the effects of HFS-induced mGluR activation with those of global mGluR activation on intrinsic excitability, we examined the effect of bath application of an agonist for group I mGluRs, DHPG, on intrinsic excitability. Intrinsic excitability was assessed by applying 1-s depolarizing current steps with varying magnitude in the presence of synaptic blockers (50 μM APV and PTX 100 μM PTX). CA1-PNs in control conditions showed tonic firing followed by AHP in response to a depolarizing current injection (Fig. 3a), and firing frequency and AHP increased as the magnitude of current increased (Fig. 3b and c). We next applied DHPG (50 μM) into the bath. Application of DHPG gradually depolarized the RMP from − 62.9 ± 1.6 mV (*n* = 7) to − 56.9 ± 1.7 mV (*n* = 7; Fig. 3d). To minimize the effect of RMP changes on spike frequency, we applied depolarizing step pulses after injecting currents to adjust the RMP to match control values (Fig. 3a). DHPG caused a significant increase in the number of APs at all applied current amplitudes (Fig. 3a and b). We also observed generation of afterdepolarization (ADP) following repetitive firing in the presence of DHPG (Fig. 3a and c). We next determined whether the DHPG effects on intrinsic excitability is mediated by mGluR5 activation. DHPG-induced changes in intrinsic excitability were reversed by mGluR5 antagonist MPEP (Fig. 3e–g), but not by mGluR1 antagonist LY367385 (Fig. 3f and g).

The difference between the effects of HFS and DHPG bath application on intrinsic excitability suggests that target ion channels underlying the effects of mGluRs are not homogenously distributed. To detect the location of mGluR5-induced changes in neuronal excitability, DHPG was applied locally by pressure application from a glass pipette into the dendritic or perisomatic region (Fig. 3h). Interestingly, only the dendritic puff application of DHPG cause the instantaneous increase in firing rates whereas the somatic puff did not trigger changes in spiking frequency (Fig. 3i). RMP depolarization was induced by DHPG application to perisomatic region, but the magnitude was variable and much smaller than that induced by DHPG bath application (Fig. 3a). Furthermore, no significant change in afterpotential was observed during DHPG local application to either perisomatic or dendritic region (Fig. 3h). Taken together, it can be suggested that significant RMP depolarization and ADP generation require global activation of mGluR5, while local activation of mGluR5 at dendritic region can sufficiently modulate AP firing by selectively targeting dendritic I_Na,P_.

**Fig. 4:**
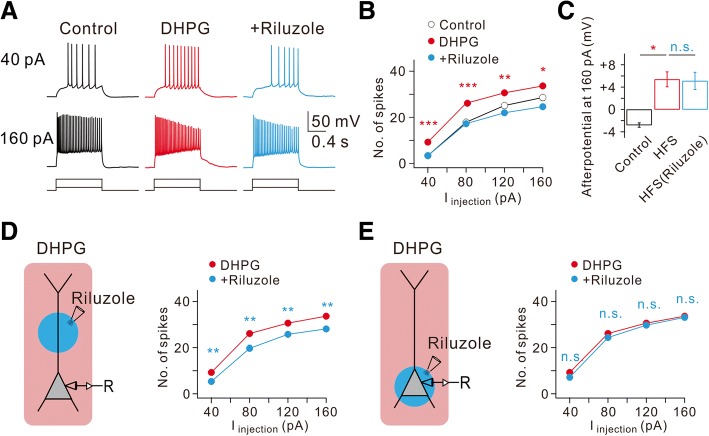
DHPG-induced increase of intrinsic excitability requires dendritic persistent Na^+^ currents. **a** Representative traces demonstrate the effects of DHPG and subsequent addition of riluzole of AP firing frequency and afterpotentials at current injections of 40 and 160 pA. **b** FI curve summarizes the effects of DHPG and subsequent addition of riluzole on AP firing frequency in response to square pulse injections. (*n* = 8, control vs. DHPG, *P < 0.05, **P < 0.01, ***P < 0.001). **c** Bar graphs summarize the effects of DHPG and subsequent addition of riluzole on afterpotentials following 160 pA square pulse injections. (*n* = 8, control vs. DHPG, *P < 0.05, n.s., *P* > 0.05). **d** and **e** Left, a schematic drawing of experimental condition demonstrates a neuron being recorded with somatic local applications of riluzole. **d** Right, FI curve summarizes the effects of DHPG and subsequent dendritic local addition of riluzole on AP firing frequency in response to square pulse injections (*n* = 8, DHPG vs riluzole, **P < 0.01). **e** Right, FI curve summarizes the effects of DHPG and subsequent somatic local addition of riluzole on AP firing frequency in response to square pulse injections (*n* = 8, DHPG vs. riluzole, n.s., *P* > 0.05)

We further examined the role of I_Na,P_ in DHPG effects using riluzole. Among DHPG effects, RMP depolarization (− 55.5 ± 1.3 mV, *n* = 4) and ADP generation were not affected by riluzole, while increased AP firing was completely reversed by riluzole (Fig. 4a, b and c). To determine the cellular location where I_Na,P_ modulation leads to increased AP firing, we employed the local puff applications of riluzole (50 μM) to the dendritic or perisomatic area in the presence of DHPG. DHPG-induced increases in AP firing was substantially inhibited by a block of dendritic I_Na,P_ (Fig. 4d), but not by block of I_Na,P_ in perisomatic regions (Fig. 4e). These results together with the effects of HFS support the idea that enhancement of dendritic I_Na,P_ mediated by group I mGluR activation is the underlying ionic mechanism of the increased AP firing.

### mGluR5-dependent Ca^2+^ release mediated by cADPR-RyR pathway underlies increased intrinsic excitability

**Fig. 5:**
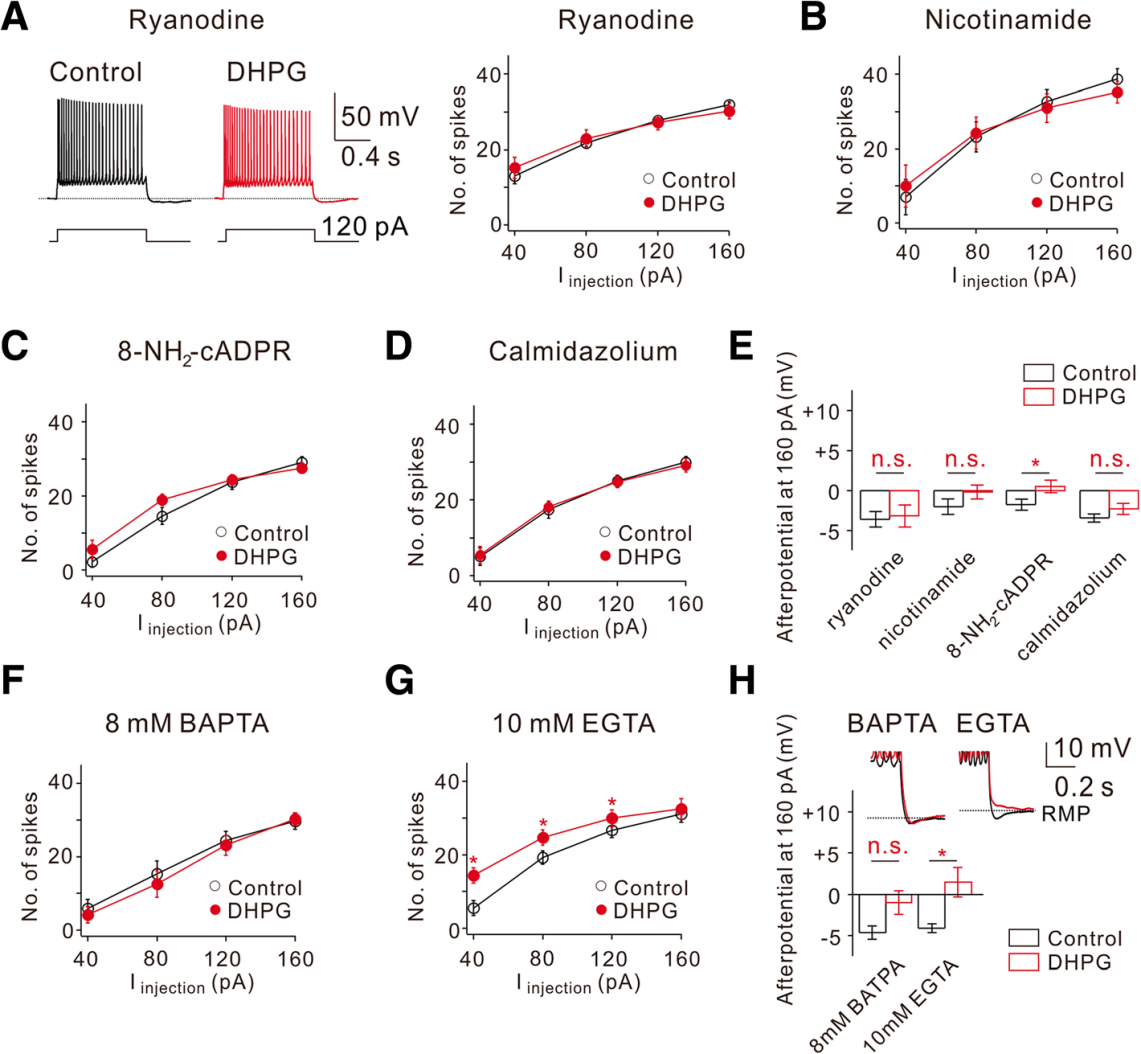
DHPG-induced increase of intrinsic excitability requires intracellular Ca^2+^. **a** Left, representative traces demonstrate the effects of DHPG on AP firing frequency and afterpotentials at 120 pA current injections when ryanodine (20 μM) was added to the pipette solutions. Right, FI curve summarizes the effects of DHPG on AP firing frequency in response to square pulse injections (*n* = 4). **b**–**d** Summary plots of the FI curve under the conditions indicated (**b**, *n* = 4, 5 mM nicotinamide; **c**, *n* = 5, 100 μM 8-NH_2_-cADPR; **d**, *n* = 5, 3 μM calmidazolium). **e** Bar graphs summarize the effects of DHPG on afterpotentials following 160 pA square current injections under different conditions indicated (n.s., *P* > 0.05, *P < 0.05). **f** and **g** Summary plots of the FI curve under the conditions indicated (*F*, *n* = 8; *G*, *n* = 11). **h** Bar graphs summarize the effects of DHPG on afterpotentials following 160 pA square current injections under different conditions indicated (8 mM BAPTA, *n* = 8, n.s., *P* > 0.05; 10 mM EGTA, *n* = 11, *P < 0.05). Note that DHPG-induced increase of after potential is abolished when 8 mM BAPTA was added to the pipette solutions

We have recently reported that mGluR5-dependent I_Na,P_ increase is mediated by Ca^2+^/calmodulin signaling that is activated by intracellular Ca^2+^ release via cADPR/ryanodine receptor (RyR) pathway [32]. However, Park et al. [20] showed that mGluR-dependent increase in R-type Ca^2+^ currents mediates ADP generation by DHPG. It is possible that different Ca^2+^ sources are involved in regulating different ion channels. To examine this possibility, we analyzed changes in intrinsic excitability under experimental conditions where cADPR/ RyR pathway was blocked. Under these conditions, DHPG-induced RMP depolarization were not significantly affected, but DHPG-induced increase in AP firing as well as DHPG-induced ADP generation were almost completely abolished (Fig. 5a to e), suggesting that these effects are dependent on cADPR/RyR-mediated Ca^2+^ release.

HFS to Shaffer collateral pathway induces Ca^2+^ release which is confined to proximal apical dendrites [32], and the effect of HFS on intrinsic excitability is selective for modulating the timing and precision of AP firing, which is mediated by I_Na,P_ enhancement (Figs. 1 and 2). By contrast, global activation of mGluRs by DHPG induces complex effects on intrinsic excitability (Fig. 3), suggesting that mGluRs target multiple ion channels. We tested the hypothesis that interaction between mGluR5 and target ion channels occurs locally in a restricted region of the neuron. To test this idea, we used Ca^2+^ buffer with different kinetics, BAPTA and EGTA. In the presence of 8 mM BAPTA, a fast Ca^2+^ buffer in the intracellular solution, DHPG effects on both AP firing and ADP were completely abolished (Fig. 5f and h), while these effects were diminished but not completely abolished in the presence of EGTA, a slow Ca^2+^ buffer (Fig. 5g and h). More powerful effects of BAPTA on inhibiting DHPG-mediated changes compared with EGTA effects suggest that the distance between the Ca^2+^ source and the target ion channels is very close within a nanometer scale so that a slow Ca^2+^ buffer is not sufficient for blocking Ca^2+^ signaling. However, DHPG-induced depolarization still occurred in the presence of BAPTA or EGTA, suggesting that the ion channel mechanisms responsible for DHPG-induced depolarization, possibly activation of TRPC channels [7] or inhibition of leak K^+^ channels [8] are not Ca^2+^-dependent.

## Discussion

In summary, we assess how mGluR5 signaling can modulate the intrinsic excitability of CA1 pyramidal neurons. Specifically, high-frequency stimulation (HFS) at CA3-CA1 synapses activates mGluR5 in the dendrites of CA1-PNs. The activation of mGluR5 induces cADPR/RyR-dependent Ca^2+^ release, which in turn, activates Ca^2+^/CaM signaling. Finally, mGluR5-mediated Ca^2+^/CaM signaling causes an increase in I_Na,P_ in the dendrites, leading to increasing firing rate and consequently improving spike-timing precision that is accompanied by decreasing the variability of onset timing. As mGluR5 activation can be induced by physiologically plausible brief high-frequency stimulation at CA3-CA1 synapses, mGluR5-induced changes in intrinsic excitability of CA1-PNs may have implications for our understanding of hippocampal memory processing.

### Dendritic persistent sodium current and advancement of spike timing

Persistent sodium currents are present in neurons throughout the central nervous system [4]. I_Na,P_, which is an inward, depolarizing current, differ from fast Na^+^ current by the lower activation threshold and non-inactivating property [4]. These features render I_Na,P_ to play crucial roles in intrinsic function of the neurons at subthreshold level, such as regulation of spike timing precision [31] and amplification of synaptic inputs [28]. Recently we demonstrated that Nav1.6 channel-mediated I_Na,P_ in CA1 pyramidal neuron dendrites causes a significant amplification of EPSPs, which induce E-S potentiation [32]. In the current study, we extend our previous findings by examining the effects of mGluR5 signaling on neuronal excitability. Our results reveal that mGluR5-mediated enhancement of dendritic I_Na,P_ increases depolarization rate during somatic current injection, resulting in the advancement of AP initiation with an increase in temporal precision (Fig. 3). These results have major implications for spatial memory and spatial navigation. When hippocampal place cells increase their firing rates while the rat approaches and passes the cell’s place field, spike timing gradually shifts to earlier phases of the theta cycle, which is known as phase precession or phase advance [19, 25]. The phase relationship of the spike to the theta cycle is a good predictor of the rat’s position in space [13, 29]. Phase precession may be caused by the increased synaptic input in the place field, but the exact amount of synaptic inputs required to produce phase advancement could be regulated by dendritic voltage-gated ion channels, such as H-channels [16]. We found that advancement of AP initiation also occurred when 5-Hz sine wave currents were injected (Fig. 2e), suggesting a possibility that enhancement of dendritic I_Na,P_ may contribute to dendritic mechanisms of phase precession [16]. Furthermore, the ability of dendritic I_Na,P_ to increase temporal precision (Fig. 3) may also have functional significance.

However, it was reported that I_Na,P_ reduces precision of spike timing evoked by single EPSPs and increases spike latency [31]. The apparent difference in the roles of I_Na,P_ in AP firing may be due to the different experimental methods for modulating I_Na,P_ in CA1-PNs. Vervaeke et al. [31] used dynamic clamp method to eliminate total I_Na,P_, whereas in our experiment, local application of DHPG or HFS at CA3-CA1 synapses selectively increases dendritic I_Na,P_. Our study highlights the unique role of increased dendritic I_Na,P_ by activating mGluR5 signaling in the proximal apical dendrites of CA1-PNs. The results of the present study are in agreement with the previous results showing that mGluR signaling targets Nav1.6 (Fig. 6 in [32]) and that Na^+^ channel subunits distributed in CA1 pyramidal neuron dendrites is Nav1.6 [15]. However, it is important to note that Nav1.6 is also concentrated at the axon initial segment (AIS) in CA1-PNs [9, 21]. Despite the abundance of a Nav1.6 expression at the AIS, selective activation of somatic mGluR5-signaling (Fig. 3h) or the local application of riluzole to the soma in the presence of DHPG in the bath (Fig. 4e) did not cause any changes in AP firing frequency. The reason why Nav1.6 in the AIS is not regulated by mGluR5 signaling remains to be examined.

### Functional implication of mGluR5-signaling

Two recent studies have found that cells with an increased intrinsic excitability before exposure to a novel environment were likely to become place cells [2, 14]. Because synaptic plasticity-inducing events are not required for the formation of place cells in novel environment [2], differences in intrinsic excitability could be the cellular substrate for place field formation in CA1-PNs [2, 14]. Generally, the intrinsic excitability is a major component determining the input-output functions, therefore, better knowledge about regulation rules of intrinsic excitability and modulatory influences that reshape the input-output function in CA1-PNs is essential for understanding the mechanism of place field formation. Our findings reveal that HFS-induced mGluR5 signaling, which induces potentiation of I_Na,P_, transiently increases the gain of input-output functions in the absence of sustained synaptic inputs, and therefore this mGluR5- activated increase in dendritic excitability may support those two in vivo evidence [2, 14] showing that modulation of intrinsic properties of CA1 pyramidal neuron dendrites determines whether a pyramidal cell become a place cell or not.

### Ionic mechanism of mGluR5-mediated effects

We demonstrated that the intrinsic excitability of CA1-PNs is greatly enhanced by DHPG. Two key effects of group I mGluR on CA1 pyramidal neuron excitability were the increased AP frequency in response to long depolarization and the generation of ADP after repetitive firing (Fig. 4). We found that both effects are almost completely abolished by inhibition of Ca^2+^ release from ryanodine-sensitive stores or CaM and intracellular BAPTA, but not by EGTA (Fig. 5). Therefore, we propose that the target channels involved in increasing firing rate and the generation of ADP are both in close proximity to RyR and regulated by local Ca^2+^. Our recent work [32] and the present results provided solid evidence that enhanced dendritic I_Na,P_ induced by either mGluR5 agonist or HFS at CA3-CA1 synapses plays a significant role in the increase in firing rate and the improvement of spike timing precision. However, mGluR5-mediated ADP generation was not occluded by the blockade of I_Na,P_ (Fig. 4c), suggesting that the frequency changes and the generation of ADP might be operated by different target ion channels, despite the shared involvement of cADPR-RyR signaling pathways (Figs. 4 and 5). Furthermore, we observed that HFS at CA3-CA1 synapses and local application of DHPG fully reproduce mGluR5-dependent frequency changes, but they fail to induce ADP generation or gradual depolarization, indicating that mGluR-dependent I_Na,P_ modulation is highly compartmentalized to the apical dendrites, while ADP generation and gradual depolarization after mGluR5 activation require global modulation of ion channels. Together, our data suggest that the impact of mGluR5 signaling on CA1-PNs are highly complex and a variety of ion channels are recruited upon their differential subcellular localizations.

## Methods

### Animals and ethical approval

All animal studies and experimental protocols were approved by the Institutional Animal Care and Use Committee (IACUC, approval No. SNU-090115-7) at Seoul National University. The animals were maintained in standard environmental conditions (25 ± 2 °C; 12/12 h dark/light cycle) and were housed under veterinary supervision at the Institute for Experimental Animals, Seoul National University College of Medicine.

### Hippocampal slice preparation

Hippocampal slices were prepared from Sprague–Dawley rats (P11–P15) of either sex. After rats were anaesthetized by inhalation of 5% isoflurane, they were decapitated and the brain quickly removed and chilled in an ice-cold high-magnesium cutting solution containing the following (in mM): 110 choline chloride, 25 NaHCO_3_, 20 Glucose, 2.5 KCl, 1.25 NaH_2_PO_4_, 1 Sodium pyruvate, 0.5 CaCl_2_, 7 MgCl_2_, 0.57 Ascorbate, with pH adjusted to 7.4 by saturating with carbogen (95% O_2_, 5% CO_2_), and with osmolality of approximately 300 mOsm/L. The isolated brain was glued onto the stage of a vibrating blade microtome (Leica VT1200) and 340 μm-thick transverse hippocampal slices were cut. The slices were incubated at 34 °C for 30 min in the artificial cerebrospinal fluid (aCSF) containing the following (in mM): 125 NaCl, 25 NaHCO_3_, 20 Glucose, 2.5 KCl, 1.25 NaH_2_PO_4_, 1 Sodium pyruvate,1 CaCl_2_, 0.5 MgCl_2_, 0.57 Ascorbate, bubbled with 95% O_2_ and 5% CO_2_., and thereafter maintained at room temperature.

### Electrophysiological recordings

Whole-cell voltage- or current-clamp recordings from hippocampal CA1-PNs (one cell per slice) were performed at 32 ± 1 °C and the rate of aCSF perfusion was maintained at 1–1.5 ml min^− 1^. The recordings were made in somata with EPC-10 amplifier (HEKA Electronik) at a sampling rate of 10–50 kHz. For current-clamp recordings, the membrane potential was held constantly at RMP. Patch pipettes (3–4 MΩ) for current clamp mode were filled with internal solutions containing the following (in mM): 130 Potassium gluconate, 20 KCl, 10 Na_2_-Phosphocreatine, 10 HEPES, 2 MgATP, 0.3 NaGTP, 0.1 EGTA. Monopolar electrodes (tip size: 5–10 μm) filled with aCSF were connected to the stimulator (Master-8, AMPI, Jerusalem, Israel) to deliver HFS. We recorded series resistance throughout experiments, and excluded neurons with series resistance > 20 MΩ from data analysis. Membrane potential values were presented without correcting the liquid junction potential. Neurons with resting potentials more positive than − 55 mV were discarded.

For local application of drugs, a pneumatic pump (Toohey Spritzer) was used with a puffing glass pipette (tip size: 1.5–2 μm). The puff area was determined from the fluorescence profile of Alexa Fluor 488 (100 μM, Invitrogen), which was added to the puffing pipette. The ejection pressure was adjusted (typically to 0.5–1 psi) such that the puff area became a circular region that diameter was 20 μm from the center (the puffing pipette tip) at 2 s after the start of ejection. Recordings obtained between 3 and 5 min after the start of drug ejection were regarded to represent drug effects.

### Drugs

(*RS*)-3,5-DHPG, LY367385, MPEP, APV, picrotoxin, ryanodine, riluzole were purchased from Tocris Bioscience. Choline chloride was from Junsei Chemical (Tokyo, Japan). 8-NH_2_-cADPR was from Molecular Probes. All other drugs were purchased from Sigma-Aldrich (St Louis, MO, USA). Stock solutions of drugs were made by dissolving in deionized water or DMSO according to manufacturer’s specifications and were stored at − 20 °C. On the day of the experiments, one aliquot was thawed and used. The concentration of DMSO in solutions was maintained at 0.1%.

### Data analysis

The amplitudes of the AHP is measured as the voltage difference between RMP and the most negative value of AHP, while the amplitude of DHPG-induced ADP was quantified in a similar manner at the same time point where we determined AHP amplitude. Expanded phase-plane plots of d*V*/d*t* indicating the shift of AP threshold were obtained from the traces shown in (Fig. 2c) which are averaged traces shown in (box, Fig. 2a). All data were presented as mean ± standard error of the mean (SEM). Statistical analysis was performed using IgorPro (version 6.1, WaveMetrics) and OriginPro (version 9.0, Microcal). Wilcoxon signed-rank test, Mann-Whitney test, and Paired-sample t-test were used. *P*-values of < 0.05 were considered statistically significant.

## Abbreviations

AMPA: α-amino-3-hydroxi-5-methyl-ioxyzole-4-propionic acid
APV: (2R)-amino-5-phosphonopentanoic acid
cADPR: cyclic adenosine diphosphate ribose
CaM: Calmodulin
DMSO: Dimethyl sulfoxide
HFS: High-frequency stimulation
I_Na,P_: Persistent sodium currents
mGluR: Metabotropic glutamate receptor
MPEP: 2-methyl-6-(phenylethynyl) pyridine
NMDA: *N*-methyl-*D*-aspartate
PTX: Picrotoxin
RS-DHPG: (RS)-3,5-Dihydroxyphenylglycine
RyR: Ryanodine receptor
SC synapse: Schaffer Collateral synapse
